# Supplementary material for: MetaRibo-Seq measures translation in microbiomes
Source: Nat Commun. 2020 Jun 29;11:3268. doi: 10.1038/s41467-020-17081-z (PMC7324362; doi:10.1038/s41467-020-17081-z)
Supplement: Supplementary file 10 — Supplementary Data 7 [file 41467_2020_17081_MOESM10_ESM.zip › File2/Confidence_High_Taxonomy/84239_out.krona.html]

Javascript must be enabled to view this page.

members
magnitude
magnitudeUnassigned
count
unassigned
taxon
rank

84239\_out

3

superkingdom
2
3

phylum
976
3

3
200643
class

3
171549
order

family
171552
3

genus
838
3

1

SRS013170\_contig\_number\_4110
species
652721

1739459
species

SRS014402\_contig\_number\_10571SRS056537\_contig\_number\_3100
2
